# Common Predisposing Factors in Mortality of Patients After Undergoing Mitral Valve Surgery at a Tertiary Care Hospital in Karachi

**DOI:** 10.7759/cureus.11964

**Published:** 2020-12-07

**Authors:** Zara Shirazi, Kashif Zia, Ali R Mangi, Syed Minhaj, Vikram Utradi, Hafeezullah Bughio, Asmatullah Achakzai, Musa Karim, Fazal Rabbi, Pervaiz A Chaudry

**Affiliations:** 1 Cardiac Surgery, National Institute of Cardiovascular Diseases, Karachi, PAK; 2 Statistics, National Institute of Cardiovascular Diseases, Karachi, PAK

**Keywords:** mitral valve replacement, mortality, predictors, common factors

## Abstract

Background

Isolated mitral valve replacement is a routinely performed procedure at our institute due to higher prevalence of rheumatic heart disease in every age category. Hardly any researches are available that dictate the predictors of surgical mortality in isolated mitral valve procedure. The aim of this study was to identify the most prevailing pre-operative factors in patients who had mortality after isolated mitral valve surgery.

Methodology

A retrospective observational study of two years was performed from January 2018 to December 2019 at the Adult Cardiac Surgery Department of a tertiary care cardiac center in Karachi, Pakistan. Patients of either gender of age ranging from 16 to 65 years who had mortality within 30 days after isolated mitral valve surgery were included in the study. Variables assessed from records were anemia, New York Heart Association (NYHA) functional classification, prolonged symptoms, poor nutritional status, degree of left ventricular (LV) dysfunction, valve pathology, pulmonary artery hypertension, and cardiac arrhythmias.

Results

We report our isolated mitral valve mortality rate of 5.5% (38/697) in the two-year duration. The most commonly encountered pre-operative factors were severe mitral regurgitation and pulmonary artery hypertension, which were observed in 32 (84.2%) and 23 (60.5%) patients, respectively. Other factors that were common to these patients were higher NYHA functional class (class III in 23 [60.5%] and class IV in 9 [23.7%]), prolonged duration of symptoms (20 [52.6%]), and right ventricular dysfunction (moderate in 21 [55.3%] and severe in 7 [18.4%]).

Conclusions

The outcome of our study suggests that severe mitral regurgitation, pulmonary artery hypertension, high NYHA functional class, LV dysfunction, and prolonged symptoms were the common predisposing factor in patients with peri-operative mortality after isolated MVR.

## Introduction

Mitral valve dysfunction (MVD) due to rheumatic heart disease (RHD) is the most frequently encountered surgical disorder in the developing world [1,2]. With the help of medical treatment, patients experiencing symptoms attain improvement, but the therapy of choice is surgical intervention, either mitral valve repair or replacement (MVR/r) [3]. The end result following MVR/r depends on the factors including pre-operative status, mitral regurgitation (MR) severity, left and right ventricular function, disease chronicity, and surgical expertise [3,4]. Patients with untreated degenerative MVD have substandard projection, with annual death rates reaching up to 34% [5]. While the untreated rheumatic MVD (rMVD) mortality rate and predictors of outcome after rMVD surgery are yet to be explored, one study from the same region has reported operative mortality of 15% in 80 patients undergoing MVR [2].

The objective of MVr in rheumatic mitral valve is to improve cardiac function and functional class and to avoid early redo surgery, as repair is usually not durable with rheumatic etiology [3,6]. Frequently associated comorbidities such as severe emphysema, restrictive lung disease, and pulmonary hypertension are usually presented with serious long-established MR and corresponding contraindication to surgical approach [7]. Aortic calcification, right ventricular (RV) dysfunction, and extreme mitral annular calcification (MAC) are considered as contraindications for MVR. Surgical risk can be determined with the help of the Society of Thoracic Surgeons (STS) risk calculation along with determination of crucial morbidity [8]. Three of the extensively utilized versions, pertinent to different cardiac approaches, include the European System for Cardiac Operative Risk Evaluation (EuroSCORE), the STS algorithms, and the Parsonnet score [7].

Due to higher prevalence of RHD in all age groups of our population, the isolated MVr is a routinely performed procedure at our institute. Very limited data are available regarding predisposing factors and predictors of surgical mortality in isolated MVr procedure for our population. Therefore, focus of this analysis was to determine the common predisposing factors in mortality of patients after MVr surgery at a tertiary care cardiac center in Karachi, Pakistan.

## Materials and methods

This retrospective observational study of two years was performed from January 2018 to December 2019 at the Adult Cardiac Surgery Department of the National Institute of Cardiovascular Disease, Karachi, Pakistan. In this study, patients of either gender, with age ranging from 16 to 65 years, who had peri-operative mortality (Peri-M) after isolated MVR surgery for stenotic, regurgitant, or mixed mitral lesions were included. MVr surgery, ischemic mitral surgery, double valve procedures including tricuspid repair, redo surgery, combined coronary with mitral valve surgery, congenital mitral valve surgery, and emergency surgery were excluded from study. Peri-M was defined as mortality from the operating room (OR) table till 30 days in-hospital after isolated MVR.

Data regarding pre-operative patient-related variables were extracted from hospital records, which included anemia (hemoglobin levels before surgery of ≤ 12 g/L for female and ≤ 13 g/L for male patients), the New York Heart Association (NYHA) functional classification (from class I to IV), age groups (16 to 20 years, 21 to 49 years, and 55 to 65 years), prolonged symptoms (for ≥ one year), poor nutritional status or hypoalbuminemia (either body mass index [BMI] ≤ 18 kg/m^2^ or pre-operative serum albumin ≤ 3.0 g/L), primary mitral valve pathology (stenotic, regurgitant, or mixed), ventricular functions (degree of right ventricular dysfunction and left ventricular ejection fraction [LVEF] ≤ 35%), pulmonary artery systolic pressure (PASP) ≥ 50 mmHg, and cardiac rhythm (permanent type atrial fibrillation with or without left atrial clot).

For the analysis of data, Statistical Package for the Social Sciences (SPSS) Version 21 (IBM Corp., Armonk, NY, USA) was used. Mean ± standard deviation was calculated for continuous variables, and categorical response variables were expressed as frequency (%). Data were obtained for analysis from hospital records after departmental as well as Institutional Ethical Review Board approval.

## Results

Overall, 697 patients underwent isolated MVR during the research period at our institute. Majority of them were females, i.e., 443 (63.6%). All of them were symptomatic and met either class I or IIa American Heart Association (AHA) guideline criteria for stenotic, regurgitant, or mixed mitral lesions. Out of these 697 patients, 38 patients had Peri-M, with a mortality rate of 5.5%. The baseline demographic and clinical characteristics of 38 patients with Peri-M after isolated MVR are presented in Table 1.

**Table 1:** Baseline demographic and clinical characteristic of 38 patients with peri-operative mortality after isolated MV surgery EF, ejection fraction; MV, mitral valve

Characteristics	Summary Statistics
Total (N)	38
Gender distribution	
Male	11 (28.9%)
Female	27 (71.1%)
Mean EF	41.84±6.91%
Type of procedure	
MV replacement	32 (84.2%)
MV repair	4 (10.5%)
Minimally invasive MV surgery	2 (5.3%)

The most common pre-operative factors were found to be severe mitral valve regurgitation and pulmonary artery hypertension. These two factors were found in 32 (84.2%) and 23 (60.5%) of patients, respectively. The mean PASP was 52.63±16.91 mmHg. Apart from these other common factors were poor NYHA functional class (class III in 23 [60.5%] and class IV in 9 [23.7%]), prolonged duration of symptoms (for ≥ one year; 20 [52.6%]), and right ventricular dysfunction (moderate in 21 [55.3%] and severe in 7 [18.4%]).

Out of these 38 patients, 1 (2.6%) patient died on the operating table due to severe right ventricular dysfunction and difficulty in weaning off cardiopulmonary bypass, 9 (23.7%) patients had severe post-operative respiratory distress with prolonged ventilation time, 11 (28.9%) patients died of low cardiac output state (LCOS), 6 (15.8%) patients had post-operative pulmonary hypertensive crisis with bi-ventricular dysfunction, and two (5.3%) patients died of peri-operative stroke, one of these had undergone large left atrial clot removal. Rest of the patients died of miscellaneous reasons such as renal dysfunction, ischemic gut complications, heparin- or drug-related complications, and fatal cardiac arrhythmias. Clinical characteristics of 38 patients with Peri-M after isolated MVR are presented in Table 2.

**Table 2:** Clinical characteristic of 38 patients with peri-operative mortality after isolated mitral valve surgery AF, atrial fibrillation; BMI, body mass index; Hb, hemoglobin; LA, left atrial; LVEF, left ventricular ejection fraction; NYHA, New York Heart Association; PASP, pulmonary artery systolic pressure

Variables	Summary Statistics
Total (N)	38
Anemia	
Female (Hb ≤ 12 g/L)	7.9% (3)
Male (Hb ≤ 13 g/L)	2.6% (1)
NYHA functional classification	
I	2.6% (1)
II	13.2% (5)
III	60.5% (23)
IV	23.7% (9)
Age groups	
16 to 20 years	7.9% (3)
21 to 49 years	81.6% (31)
50 to 65 years	10.5% (4)
Prolonged symptoms	
≥ 1 year	52.6% (20)
Mean duration of symptoms	8.1 ± 5.9 years
Poor nutritional status	
BMI ≤ 18 kg/m^2^	13.2% (5)
Serum albumin ≤ 3.0	7.9% (3)
Primary mitral valve pathology	
Severe stenosis	5.3% (2)
Severe regurgitation	84.2% (32)
Mixed lesion	10.5% (4)
Ventricular function	
Normal	7.9% (3)
Mild	18.4% (7)
Moderate	55.3% (21)
Severe	18.4% (7)
LVEF less than 35%	34.2% (13)
PASP	
Mean PASP (mmHg)	52.63 ± 16.91
PASP ≥ 50	60.5% (23)
Cardiac rhythm	
Sinus rhythm	76.3% (29)
Atrial fibrillation	15.8% (6)
AF with LA clot	7.9% (3)

## Discussion

The aim of our study was to determine the factors that may predict in-hospital mortality in RHD patients who have undergone isolated MVr. Mitral stenosis and/or regurgitation is one of the most usual valvular problems of RHD [9]. In most of the cases, MVr is generally necessary; however, in few cases, MVR can also be executed [9]. There is a higher prevalence of operative mortality associated with MVr [10]. Therefore, our research specifically gives importance to common variables that could have a notable influence on operative mortality in MVR/r surgery [10].

For mitral valve pathology caused due to degenerative changes, surgical repair or replacement is the main management option. There are countless elements on which patient’s end results depend, which included pre-operative status, severity of MR, anemia, symptoms, nutritional status, echocardiographic findings, the approach of repair, and surgeon and center experience.

In our study, majority were females of reproductive age category. A large number of patients had a substandard functional class (NYHA class III and IV), which denotes delayed presentation of disease. The patient’s age was one of the factors. Age is recognized as a possible factor for mortality in rheumatic valve replacement patients; however, the age at which death rate escalated is much lower for rheumatic population. Anemia was among the finding of the patients and is an established predictor of surgical mortality.

It is a concern for additional studies whether the rise in surgical deaths is because of severity of the disease or due to hidden intraoperative thromboembolic episodes. Hellgren et al. have reported poor NYHA class, but not older age, as a possible factor for pre-mature mortality [11]. Poor nutritional status or hypoalbuminemia was also noted as one of the factors in our study. Left ventricular dysfunction has been reported as a significant predictor of short- and long-term outcomes of the patients.

Patients with degenerative mitral valve disease who develop symptoms of MR have a bad prognosis, with yearly death rates of up to 34% [3]. Mitral stenosis, mainly due to RHD, is usually managed by percutaneous balloon mitral valvuloplasty or MVR. Repair is generally not practicable in patients with rheumatic mitral disease. While high-grade primary MR still receives management with surgical intervention, percutaneous techniques for repair and replacement are also gaining traction. MVR may be considered in patients with MR caused by papillary muscle rupture or degenerative and ischemic MR, or in patients with a failed repair undergoing reoperation.

Not doing surgical correction till symptoms arise is troublesome since the end results may be substandard at that period, with a maximum chance of postoperative left ventricular dysfunction and death. Upcoming researches should have center of attention on the important independent predictors of long-term mortality after MVr. Moreover, satisfaction toward life and health issues should also be acquired after MVr. Even so, this study has registered down some of the notable potential predictors of mortality after MVr in developing countries.

The main limitation of this study was a lack of comparative control group, and, secondly, the retrospective nature of the research, due to which data for some of the important parameters such as left ventricular volume, left ventricular volume, and pre- and post-operative renal dysfunction were not available. The fundamental restriction in the prediction of end result in patients undergoing cardiac surgery is the phenomenon of extensive array of complications due to multifariousness interaction between surgery and anesthesia. Lastly, analysis was limited to the available in-hospital outcome data. Additional studies with longer follow-up after surgeries will illustrate long-term predictors of mortality in these patients.

## Conclusions

The conclusion of our study is that valve pathology such as severe regurgitation, pulmonary artery hypertension, poor NYHA functional class, left ventricular dysfunction, and long duration of symptoms were the common predisposing factor in patients with Peri-M after isolated MVR. Nevertheless, additional long-term clinical investigations with larger specimens should be directed to decide predictors of mortality after replacement of mitral valve in progressing countries.
